# Theca lutein cysts and early onset severe preeclampsia

**DOI:** 10.11604/pamj.2016.24.141.7247

**Published:** 2016-06-14

**Authors:** Mehmet Akif Sargin, Niyazi Tug, Ozgur Aydin Tosun, Murat Yassa, Evrim Bostanci

**Affiliations:** 1 Department of Obstetrics and Gynecology, Fatih Sultan Mehmet Research and Training Hospital, Istanbul, Turkey; 2 Department of Obstetrics and Gynecology, Zeynep Kamil Research and Training Hospital, Istanbul, Turkey; 3 Department of Obstetrics and Gynecology, Sincan State Hospital, Ankara, Turkey

**Keywords:** Hydrops fetalis, hyperreactio luteinalis, severe preeclampsia

## Abstract

Hyperreactio luteinalis (HL) is a rare condition that is characterized by bilateral ovarian enlargement and multiple thin walled cysts. Hypersensitivity of the ovary to circulating human chorionic gonadotropin (hCG) is playing the main role in pathophysiology. HL observed in cases where there is high serum ß-HCG levels, similarly to gestational trophoblastic disease, multiple pregnancies, hydrops fetalis and after fertiliy treatment. Most of HL are self limiting condition and patients are asymptomatic. Differentiation from ovarian malignancies is important. This is a case report of severe preeclampsia prior to 20 weeks gestation due to hyperreactio luteinalis.

## Introduction

Hyperreactio luteinalis (HL) is a rare condition that is characterized by bilateral ovarian enlargement and multiple thin walled cysts. Spontaneous regression after delivery is usually seen in this condition. The underlying pathophysiologic process of HL is the hypersensitivity of the ovary to circulating human chorionic gonadotropin (ß-HCG). HL is offen associated with high maternal serum levels of ß-HCG and hyperandrogenic state. The diagnosis was often made incidentally with extra-ovarian symptoms including maternal virilization. HL can occur during spontaneous pregnancy although commonly seen in patients with high serum ß-HCG levels similarly to gestational trophoblastic disease, multiple pregnancies, hydrops fetalis and after fertiliy treatment. However more than half of the reports occurred with normal singleton pregnancies [1]. Most of HL are self limiting condition and patients are asymptomatic. Thus, treatment includes non-surgical expectant management. Nevertheless, emergency surgery can be needed due to ovarian torsion [2]. Differentiation from ovarian malignancies is important. HL can mimic ovarian mucinous borderline tumor of intestinal type [3]. Obstetric complications can be seen during pregnancy with HL due to high maternal serum ß-HCG levels. The most important complications are severe preeclampsia, HELLP syndrome, intrauterine growth restriction (IUGR), hyperemesis gravidarum, hydrops fetalis, maternal hyperthyroidism, fetal and maternal virilization [4, 5]. We present a HL case that complicated with severe preeclampsia prior to 20 weeks gestation.

## Patient and observation

**Figure 1:**
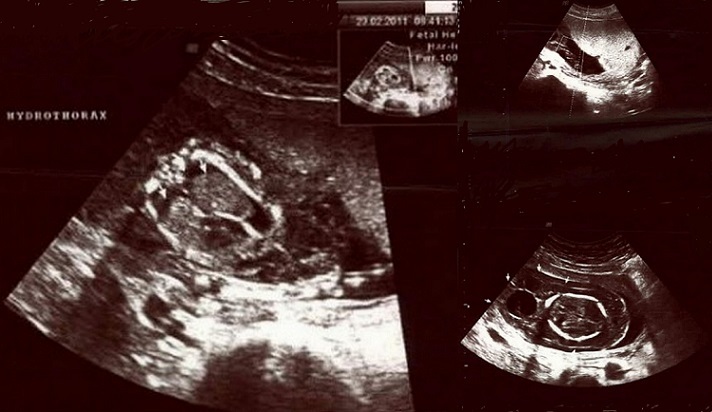
Cystic hygroma, fetal hydrothorax and abnormal umblical artery pulsed Doppler

**Figure 2:**
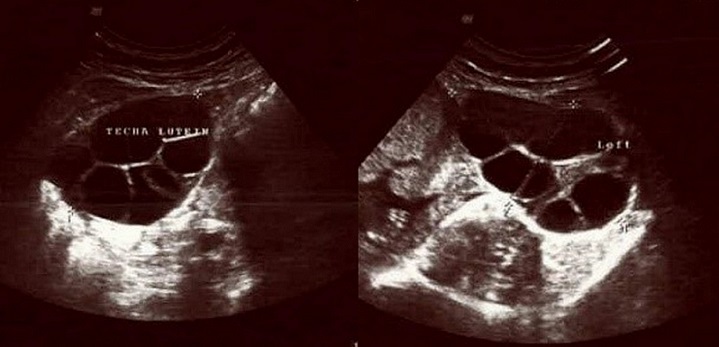
Bilateral theca lutein cysts

A 27-years-old gravida 3, para 1, abort 1 pregnant woman was referred to the emergency department of Zeynep Kamil Gynecologic and Pediatric Training Research Hospital with headache, pruritus, nausea and vomiting. Her menstrual periods were regular and first pregnancy had been uneventful. Gestational age was 18 weeks that calculated by last mentruel period day and using previous ultrasound records. On arrival her blood pressure was 180/120 mmHg. There was no history of pre-existing hypertension or renal disease and she had no other cardiovascular disease history. Her blood group was A Rh positive with no prior history of blood transfusion. Ultrasound scan showed a 18 weeks fetus with hydrops (ascites, hydrothorax and skin edema), cystic hygroma (Figure 1) and hydropic placenta. Pulsed Doppler findings were abnormal at the umbilical artery (Figure 1), the uterine artery and the fetal middle cerebral artery. No free fluid was found in the abdomen. In addition, bilateral multiseptated cystic adnexal masses that measured 7x10 cm and 10x12 cm diameters were detected (Bilateral theca lutein cysts) (Figure 2). The first laboratory examination revealed as follows: haemoglobin 11.4 g/dl; haematocrit 35.7%; platelets 199.000 per ml; blood urea nitrogen (BUN) 10 nmol/L; serum creatinine 0.5 mg/dl; aspartate aminotransferase 33 U/L; alanine aminotransferase 30 U/L and lactate dehydrogenase 243 U/L. Total and direct bilirubin levels were high as 1.2 and 0.8 mg/dl, respectively. 3+proteinuria was found on a dipstick urinalysis. A 24-h urine collection was begun to quantify proteinuria. Serum ß-hCG value was 431.336 mIU/ml. Thyroid function tests at that stage revealed hyperthyroidism with low serum TSH and elevated free T4 and T3 concentrations which are two times greater than normal ranges. Serum total testosterone (220 µg/mL) (normal, 60-150 µg/mL) and androstenedione (10 ng/mL) (normal, 0.9-3.5 ng/mL) levels were higher than normal ranges. Serum tumour markers were measured in order to exclude malignancy and no increase was found.

Magnesium sulfate therapy consisted of a continuous intravenous dose of 2 g per hour after the initial 6g loading dose, for the prevention of eclampsia. Maternal vital signs, input and output monitored closely. Concurrently, 200 mcg tablets of misoprostol inserted into the posterior fornix after the decision of pregnancy termination due to fetal anomaly and severe preeclampsia. Dose was repeated 2 hourly by orally until medical abortion. Antihypertensive therapy with methyldopa 500 mg three times daily was started. Eight hours later, she delivered a female fetus weighing 350 g with hydropic placenta weighing 450 g which had gross congenital abnormalities.

Fetal skin biopsy performed for karyotype analysis. After delivery, 2 g per hour maintenance dose of magnesium sulfate was continued until the patient fulfilled clinical criteria for discontinuation of seizure prophylaxis. Significant and severe proteinuria were identified on 24-hour urine evaluation (12 gr / 24 hours). After the first day of hospitalization, her blood pressure measured as 150/100 mmHg. Maternal vital signs were in normal limits on postpartum fifth day. Repeated laboratory tests revealed normal haemogram and biochemistry. Serum TSH, free T4 and T3 levels were tended to return to normal levels. An autoimmune screen including anticardiolipin antibodies, lupus anticoagulant, and anti-ß2 glycoprotein revealed no abnormality. Serum ß-hCG value was 21183 mIU/ml. Total proteinuria was less than 4 g in repeated 24-hour urinary collection. Patient discharged with these results. At fifteenth and thirtieth days; blood pressure, haemogram, biochemistry, 24-h urine collection, thyroid function tests and serum ß-hCG measurements were repeated. She was normotensive without medication. All of the results were in normal limits except 24-hour urine evaluation (1600 mg and 460 mg proteinurea respectively) and serum ß-hCG levels (3260 and 220 mIU/ml). Ultrasonography examination performed at postpartum sixth week that showed the ovaries had again returned to normal size and architecture. Fetal skin biopsy revealed normal fetal karyotype (46,XX). Histopathology of the placenta confirmed an accelerated villous maturation and hypermature appearance with syncytial knot formation. There was no histological changes of complete and partial moles.

## Discussion

HL is a rare, benign condition with bilaterally enlarged ovaries containing multiple theca lutein cysts and spontaneous regression after termination of pregnancy or delivery. HL could be lead to obstetric complications such as hyperthyroidism, hyperemesis gravidarum, hyperandrogenism, nonimmune fetal hydrops, IUGR, preeclampsia/eclampsia and HELLP syndrome. Our patient was symptomatic with hyperemesis gravidarum and severe preeclampsia.

Severe preeclampsia rarely occurs prior to 20 weeks of gestation except in pregnancies with triploidy [6] and maternal anti-phospholipid syndrome [7]. Approximately 35% of triploid pregnancies will go on to develop preeclampsia in the second trimester because of elevated hCG levels and/or placentomegaly. In literature, preeclampsia before 20 weeks of gestation was published as case reports that caused by anti-phospholipid syndrome and molar pregnancy. This is the first case in literature of confirmed severe preeclampsia at 18 weeks of gestation age with elevated hCG levels, fetal anomaly (cystic hygroma and nonimmune hydrops), placentomegaly and abnormal pulsed Doppler findings that was not associated with triploidy, trophoblastic disease or anti-phospholipid syndrome.

Literature review about hyperreactio luteinalis and severe preeclampsia showed that most cases with severe preeclampsia and HELLP syndrome occurs at the third trimester of pregnancy. Masuyama et al. reported severe preeclampsia associated HL at 16 weeks gestational age with normal-sized fetus, and normal pulsed Doppler findings [4]. Elevated hCG levels and bilaterally enlarged multicystic ovaries had been diagnosed in the first trimester before occurrence of preeclampsia. Follow-up of the patient with aspirin and heparin treatment continued until 32 week´s gestational age. Grgic O. et al. reported case developed IUGR, severe preeclampsia and HELLP syndrome respectively 30, 33 and 34 weeks of gestation [5]. In the first trimester, they performed unilateral laparoscopic salpingo-oophorectomy due to the ovarian torsion. Cavoretto P. et al. reported HL case at 21 gestational weeks [8]. Diagnosis was made by exploratory laparoscopy to exclude ovarian torsion and malignancy. IUGR and mild preeclampsia occurred at 34 weeks.

Hyperandrogenism is one of characteristic of HL. Serum total testosterone and androstenedione levels were significantly increased in our case. In literature review, virilization occurs most frequently in third trimester [3], and rarely in second trimester [9]. In our case, there was no any clinical sign of hyperandrogenemia such as maternal virilization due to the early termination of pregnancy.

Nonreassuring fetal status, variable and prolonged fetal heart rate deceleration, soft tissue dystocia are common indications for emergency cesarean delivery whose pregnancies complicated by HL [3–5]. Elective cesarean delivery may also be planned [9].

Mirror syndrome (Ballantyne syndrome) is a rare disorder and refers to the association of fetal hydrops and maternal heavy preeclampsia which disappears shortly after successful treatment of pregnancy termination or delivery [10]. We assessed our case as HL because of the fact that having high serum hcg and androgen levels and bilateral theca lutein cysts in ultrasonographic evaluation. Mirror syndrome and HL are described clinically different in literature. Ultrasonographic adnexial condition, serum hcg and androgen levels are not mentioned in that cases concerning Mirror syndrome. Comparison of hemogram, urinalysis, serum biochemistry, serum androgen levels and adnexial pathologies between Mirror syndrome and Hyperreactio Lutealis may indicate any connection in both cases. Moreover, such studies may establish a staging or differential system for these syndromes.

## Conclusion

In conclusion, we have presented a spontaneous single pregnancy with HL and severe early onset preeclampsia syndrome at 18 weeks of gestation. Most of HL are self-limiting condition and patients are asymptomatic. Rarely serious obstetrical complications such as severe preeclampsia and HELLP syndrome can be occur. HL is one of the causes of preeclampsia before the twentieth week. We suggest that ultrasonography scan during the first trimester for both of the ovaries must be examined carefully. Clinicians should be aware of the obstetrical complications in cases where theca lutein cysts are detected, particularly heavy preeclampsia.
